# Correlation of angiogenic biomarker signatures with clinical outcomes in metastatic colorectal cancer patients receiving capecitabine, oxaliplatin, and bevacizumab

**DOI:** 10.1002/cam4.71

**Published:** 2013-03-06

**Authors:** Yingmiao Liu, Mark D Starr, Anuradha Bulusu, Herbert Pang, Nan Soon Wong, Wanda Honeycutt, Anthony Amara, Herbert I Hurwitz, Andrew B Nixon

**Affiliations:** 1 Department of Medicine, Duke University Medical CenterDurham, North Carolina; 2 Department of Biostatistics and Bioinformatics, Duke University Medical CenterDurham, North Carolina; 3 OncoCare Cancer Centre, Mount Elizabeth Medical CentreSingapore

**Keywords:** Angiome, bevacizumab, biomarker, chemotherapy, colorectal cancer

## Abstract

A novel combination of capecitabine, oxaliplatin, and bevacizumab was evaluated in colorectal cancer patients enrolled in a phase II clinical trial. In this retrospective analysis, plasma samples from patients receiving capecitabine, oxaliplatin, and bevacizumab were analyzed to investigate biomarkers of clinical benefit. Forty-one protein biomarkers were tested in 38 patients at baseline and after two cycles of drug administration. Correlations among analytes were evaluated by Spearman analysis. Analyte levels at baseline and changes on-treatment were correlated with progression-free survival (PFS) and overall survival (OS) by univariate analysis. Multivariate analyses were determined using the Cox proportional hazard model. Time to event analyses were evaluated by Kaplan–Meier analysis and compared by log-rank test. Baseline levels of vWF and Ang-2 significantly correlated with PFS, while levels of VCAM-1, vWF, TSP-2, IL-8, MMP-2, and Ang-2 correlated with OS (*P* < 0.05). The fold change of IGF-1 levels from baseline to the end of cycle 2 was correlated with PFS, while fold changes of Ang-2, TSP-2, and TGF-β2 correlated with OS. A baseline signature of Ang-2, IGFBP-3, IL-6, and VCAM-1 identified a low-risk and high-risk group of patients (OS: 33.9 months vs. 18.1 months, respectively, *P* = 0.016). For treatment-related changes, a signature consisting of Ang-2, E-Cadherin, IL-6, MCP-1, OPN, and TGF-β1 was able to stratify patients into high- and low-risk groups (PFS: 7.7 months vs. 15.5 months, *P* = 0.004). Multiplex analysis of patient plasma in this trial identified several baseline- and treatment-related biomarkers associated with clinical outcome. These findings merit further exploration in larger, controlled clinical trials.

## Introduction

Colorectal cancer (CRC) is the second leading cause of cancer-related deaths in the United States 1. First-line chemotherapy has been shown to improve clinical outcomes, with the median overall survival (OS) of patients with metastatic CRC now approaching 20–24 months 2–4.

Angiogenesis is a critical process promoting CRC development and metastasis 5. Bevacizumab is a monoclonal antibody that specifically binds the angiogenic factor VEGF-A, thereby inhibiting downstream signaling. When given with chemotherapy, bevacizumab has been shown to significantly improve clinical outcomes for mCRC patients, in both first- and second-line settings 2,3,6. However, for many patients, the benefit from bevacizumab is modest and virtually all patients will progress on bevacizumab-based therapy 7,8. As a result, biomarkers that can identify or “predict” which patients are most likely to benefit from bevacizumab therapy are urgently needed. However, despite almost over a decade of clinical development for anti-VEGF therapies, biomarkers to predict those patients most likely to benefit or not benefit have remained elusive 9.

To explore candidate blood-based biomarkers, we have optimized a multiplex array of more than 40 angiogenic factors for use in cancer patients. Compared with biomarker analysis of tumor samples, plasma samples have the advantages of minimal risk, reduced cost, and availability in essentially all patients, both at baseline as well as along the continuum of care. Biomarker analyses of plasma samples by our group from patients with metastatic pancreatic cancer treated on CALGB80303, a phase III study of gemcitabine with or without bevacizumab, identified several markers associated with prognosis independent of treatment as well as markers associated with benefit or lack of benefit from bevacizumab specifically 10.

In the current study, we retrospectively analyzed 38 CRC patients who received capecitabine, oxaliplatin, and bevacizumab on a 2-week per cycle schedule 11. This study reported a median progression-free survival (PFS) of 10.3 months and a response rate of 52.6%. To explore potential markers of clinical benefit, we tested 41 biomarkers for all patients at baseline, as well as after two cycles of treatment.

## Materials and Methods

### Patient selection, treatment, and outcome

The treatment regimen and clinical outcomes for this study have previously been reported 11. Written informed consent was obtained from each patient regarding the use of plasma for this correlative analysis. This study was Institutional Review Board (IRB) approved and registered with http://www.clinicaltrials.gov (study number: NCT00416494). Table 1 lists the patient characteristics for the biomarker sub-population as well as the study as a whole.

**Table 1:** Characteristics of all patients on study, as well as the subpopulation whose plasma were available for biomarker analysis

Characteristic	All patients (*n* = 50)	Patients for biomarker analysis (*n* = 38)
Age, median (range)	55 (24–81)	53.5 (24–81)
Sex, *n* (%)		
Male	27 (54)	21 (55)
Female	23 (46)	17 (45)
Race, *n* (%)		
Caucasian	38 (76)	30 (79)
African American	7 (14)	5 (13)
Others	5 (10)	3 (8)
ECOG at baseline, *n* (%)		
0–1	48 (96)	37 (97)
2	2 (4)	1 (3)
PFS, median (95% CI)	10.3 (7.5–12.7)	12.7 (9.7–18.1)
OS, median (95% CI)	23.3 (14.3–31.8)	24.9 (14.4–33.4)

ECOG, Eastern Cooperative Oncology Group; PFS, progression-free survival; CI, confidence interval; OS, overall survival.

### Plasma collection, handling, and storage

In brief, at baseline and end of cycle 2, blood was collected from each patient by venipuncture into a sodium citrate vacutainer (BD Biosciences, San Jose, CA; catalog #369714), and mixed thoroughly. Additional samples were collected from patients at other time points; however, due to the inconsistent nature of collection, no analyses of time points other than baseline and end of cycle 2 were conducted. After mixing, the tubes were centrifuged at 2500*g* for 15 min. The upper layer of plasma was transferred to a fresh tube and centrifuged one more time at 2500*g* for 15 min. The double-spun, platelet-poor plasma was aliquoted, snap frozen, and stored at −80°C until use.

### Multiplex and ELISA assays

All biomarkers were measured using the SearchLight multiplex platform (Aushon Biosystems, Inc., Billerica, MA; Table 2), except for collagen-IV (Exocell Inc., Philadelphia, PA), IGF-1 (Immunodiagnostic Systems Inc., Scottsdale, AZ), CSF-1 (R&D Systems, Inc., Minneapolis, MN), and TGF-β R3 (R&D Systems, Inc., Minneapolis, MN).

**Table 2:** Levels of biomarkers at baseline and on-treatment

Biomarkers	Baseline		On-treatment			*P*-value
Median	Range	Median	Range	% BL
Ang-2 (pg/mL)	233.3	104–1334.5	191.4	88.7–841.2	76.5	<0.0001
Collagen-IV (pg/mL)	98.8	35.1–440.2	75.8	31.3–346.9	72.1	<0.0001
CRP (μg/mL)	7.1	0.1–144.4	4.6	0.14–67.3	36.1	0.0356
CSF-1 (pg/mL)	369.9	84.2–657.6	346.4	89.4–653.4	93.7	0.355
d-dimer (μg/mL)	22.2	2.0–34.5	23.9	9.1–37.2	108.7	0.009
E-Cadherin (ng/mL)	18.2	10.5–55.7	14.7	1.6–28.2	69.1	<0.0001
E-Selectin (ng/mL)	39.3	14.7–85.7	29.5	6.8–76.4	81.7	<0.0001
FGF-β (pg/mL)	11.8	0.9–162.7	12.3	1.2–94.4	100	0.6641
GRO-α (pg/mL)	24.5	9.1–110.2	19.7	8.7–53.1	91.5	0.0119
HGF (pg/mL)	524.1	174.6–4499.9	571.6	166.8–5762	106.5	0.5737
ICAM-1 (ng/mL)	365.2	113.2–1716.8	326.1	104.7–559.4	94.7	0.1737
IGF-1 (pg/mL)	58.7	19.3–150.7	76.5	25.1–179.9	130.4	0.0009
IGFBP-1 (ng/mL)	6.2	0.8–211.4	3.7	0.7–110	64.9	0.0011
IGFBP-3 (ng/mL)	565.3	287.7–809.3	585.9	244.8–1049.1	108.5	0.1035
IL-6 (pg/mL)	3.4	0–219.3	2.6	0.1–57.5	83.2	0.2859
IL-8 (pg/mL)	53.7	17.3–415.7	31.7	10.9–160.7	57.1	0.0001
MCP-1 (pg/mL)	510.8	155–2040	413.3	72.5–955	86.6	0.0249
MMP-2 (ng/mL)	308.0	198.7–415.4	368.3	265.2–552.5	122.5	<0.0001
MMP-9 (ng/mL)	34.9	10.7–299.0	34.6	5.2–323.8	104.6	0.7925
OPN (ng/mL)	62.2	5.5–87.0	57.0	16.8–90.1	96.3	0.4713
PAI-1 active (ng/mL)	1.6	0.3–10.0	1.2	0.2–10.2	72.4	0.027
PAI-1 total (ng/mL)	30.8	4.7–93.8	15.8	3.5–72.1	65.9	0.0002
PDGF-AA (pg/mL)	209.8	3.8–749.1	123	0.5–838.7	66.9	0.0086
PDGF-BB (pg/mL)	62.9	17.9–474.7	35.5	12.3–479.4	65.5	0.003
PEDF (μg/mL)	1.1	0.4–2.0	1.3	0.6–2.0	117.2	<0.0001
PIGF (pg/mL)	9.2	3.3–18.9	17.4	6.9–41.8	197.7	<0.0001
P-Selectin (ng/mL)	47.0	6.9–108.7	44.6	10.9–110.9	86.4	0.0061
SDF-1 (pg/mL)	744.2	128.2–2903.6	683.3	220.4–4344.7	125.5	0.0704
TF (pg/mL)	29.1	6.1–139.3	31	8.2–197.5	104.5	0.9038
TGF-β1 (ng/mL)	18.0	4.1–49.4	13.3	5.3–40.9	81.3	0.0143
TGF-β2 (ng/mL)	1.8	0.2–5.7	1.3	0.4–4.9	78.5	0.0429
TGFβ-R3 (ng/mL)	5.3	0.8–14.7	5.7	0.5–17.3	106.9	0.9943
TSP-1 (ng/mL)	10.0	0.2–281.6	5.6	0.3–661.3	64.3	0.11
TSP-2 (ng/mL)	17.4	4.6–112.3	18.0	6.2–60.7	74.0	0.0001
VCAM-1 (μg/mL)	1.0	0.6–2.1	1.4	0.3–2.9	132.2	<0.0001
VEGF (pg/mL)	78.3	29.1–1587.3	109.3	4.0–1245.4	109.0	0.1392
VEGF-C (pg/mL)	865.9	453.7–5229.9	811.8	402.6–4807.3	92.0	0.0446
VEGF-D (pg/mL)	829.4	359.4–12722.2	914.0	359.3–13827.9	111.3	0.0011
sVEGF-R1 (pg/mL)	121.1	17.9–4247.9	125.7	7.2–4796.1	83.1	0.5259
sVEGF-R2 (ng/mL)	5.3	3.3–46.7	4.7	2.6–7.5	91.5	0.0055
vWF (U/mL)	11.3	2–140	10.7	2.0–89.3	97.6	0.0918

Ang-2, angiopoietin-2; CRP, C-reactive protein; CSF-1, colony-stimulating factor-1; FGF-β, fibroblast growth factor basic; GRO-α, growth-related oncogene-alpha; HGF, hepatocyte growth factor; ICAM-1, inter-cellular adhesion molecule-1; IGF-1, insulin-like growth factor-1; IGFBP, insulin-like growth factor binding protein; IL-6, 8, interleukin-6, 8; MCP-1, monocyte chemotactic protein-1; MMP-2, 9, matrix metallopeptidase-2, 9; OPN, osteopontin; Pai-1, plasminogen activator inhibitor-1; PDGF-AA, BB, platelet-derived growth factor-AA, BB; PEDF, pigment epithelium-derived factor; PlGF, placenta growth factor; SDF-1, stromal cell-derived factor-1; TF, tissue factor; TGF, transforming growth factor; TSP-1, 2, thrombospondin-1, 2; VCAM-1, vascular cell adhesion molecule-1; VEGF, vascular endothelial growth factor; sVEGF-R1, 2, soluble VEGF receptor-1, 2; vWF, von Willebrand factor.

Multiplex assays were done in a 96-well format according to the SearchLight protocol. Briefly, samples were thawed on ice, centrifuged at 20,000*g* for 5 min to remove any residual precipitate and appropriately diluted before placement onto SearchLight plates. Samples and standards were incubated at room temperature for 1 h while shaking. Plates were washed three times using an automated plate washer (Biotek Instruments, Inc., Model ELx405, Winooski, VT), the biotinylated secondary antibody was added, and the plates were then incubated for an additional 30 min. After three more washes, streptavidin-HRP was added to the plates, the plates were incubated for 30 min, washed again, and SuperSignal substrate was added. Images of the plates were taken within 10 min, followed by image analysis using SearchLight array analyst software (Version 2.1). Commercial enzyme-linked immunosorbent assay (ELISA) kits were used to measure collagen IV, IGF-1, CSF-1, all according to the individual manufacturers' instructions.

Analyte concentrations were calculated based on a standard curve derived by performing four serial dilutions of the corresponding protein standard on each plate. Patient samples were tested in triplicate, and the mean value was used for analysis. Three analytes interferon-gamma (IFN-γ), N-terminal prohormone brain natriuretic peptide (NT-proBNP), tumor necrosis factor-alpha trimer (TNF-α trimer) were excluded from statistical analysis because greater than 10% of the samples fell out of the detectable range. When out-of-range values were imputated, the median value for that analyte was substituted.

For the TGF-β R3 ELISA assay, capture antibody (R&D systems, cat: AF-242-PB) was immobilized onto an EIA/RIA plate (Corning, cat: 3590) overnight. Plates were then washed, samples were loaded, and the plates were incubated at room temperature for 2 h. Then detection antibody (R&D systems, cat: BAF-242) was applied and the plates were incubated for 2 h, followed by the addition of streptavidin-horseradish peroxidase (HRP) (R&D systems, cat: DY998) and again incubated for 30 min. Finally, Fast OPD substrate (Sigma, cat: P9187) was added, 3 mol/L HCl was applied to stop reaction 30 min later, and optical absorbance at 490 nm was recorded immediately.

### Statistical analysis

To evaluate on-treatment changes, L-ratio was calculated using the formula Log_2_(posttreatment level/baseline level) for each analyte. To determine the significance of L-ratio changes, signed-rank tests were conducted and *P*-values were shown on Table 2. Waterfall plots were produced for L-ratios to display changes between the time points. Spearman correlations were calculated for all pairs of analytes at both baseline and L-ratio. Hierarchical clustering was used to group the analytes, which were displayed as dendrograms.

Univariate Cox proportional hazards analysis was performed for each analyte for both PFS and OS. Multivariate analysis was performed with Cox proportional hazards model to generate baseline and on-treatment biomarker signatures. For feasibility reasons, the number of biomarkers included in any model was limited to be 10 or less. We utilized the score selection method to control the number of analytes in the model 12; leave-one-out cross-validation was used to derive the best prognostic signature 13. Kaplan–Meier plots were used to illustrate patients' survival, and log-rank tests were applied to test the predictive value of each signature.

## Results

### Changes in biomarker levels in response to capecitabine, oxaliplatin, and bevacizumab

All patients received a combination of chemotherapy (capecitabine and oxaliplatin) as well as bevacizumab in this single-arm, nonrandomized study. To evaluate biomarker responses to treatment, each patient's baseline biomarkers profile was used as his or her reference control. Plasma samples collected at baseline (BL) and at the end of cycle 2 (on-treatment) were available for biomarker analysis from 38 of the 50 patients treated on the parent study. The biomarker population was similar to the overall study population, regarding age, gender, race, and ECOG performances (Table 1). For the parent study, the median PFS, median OS, and response rate were 10.3 months (95% CI, 7.5–12.7), 23.3 months (95% CI, 14.3–31.8), and 50%, respectively 11. In the subpopulation of 38 patients whose samples were available for biomarker analysis, median PFS, median OS, and response rate were 12.7 months (95% CI, 9.7–18.1), 24.9 months (95% CI, 14.4–33.4), and 52.6%, respectively (Table 1).

**Figure 1:**
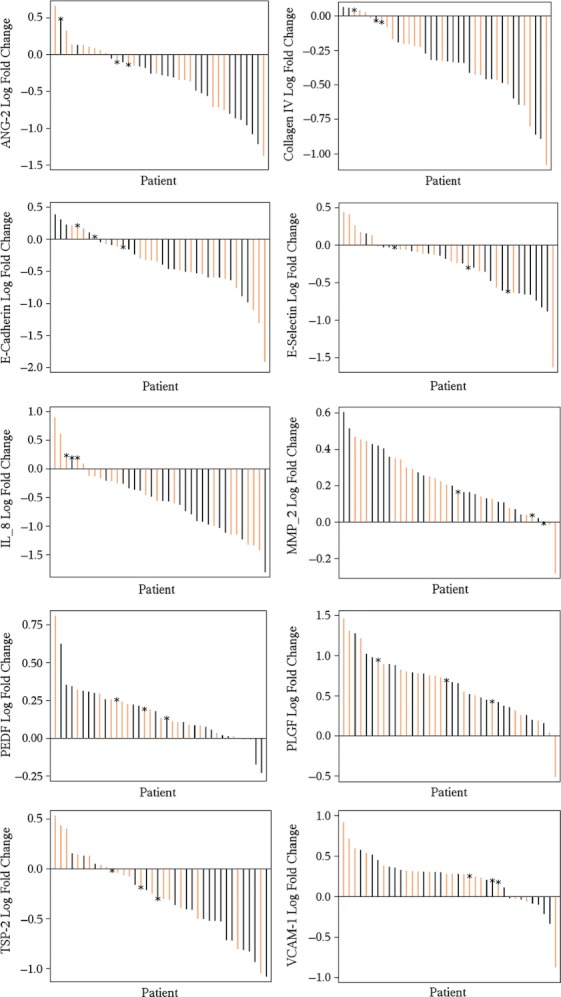
Change from baseline to the end of cycle 2 for biomarkers with statistical significance (*P* ≤ 0.0001). *Censored patients. Golden lines represent patients whose progression-free survival (PFS) ≥ median; black lines represent patients whose PFS < median.

In total, 41 biomarkers for each patient were analyzed at both baseline and on-treatment. The median level and range for each biomarker is shown in Table 2. Assays were highly reproducible with coefficient of variation generally in the 5–20% range (data not shown). As shown in Table 2, the statistically significant changes were observed in Ang-2, Collagen IV, E-Cadherin, E-Selectin, IL-8, MMP-2, PEDF, PlGF, TSP-2, and VCAM-1 (*P* ≤ 0.0001). It should be noted that the percent change from baseline (% BL) reflects the average of each individual patient's change across all patients. Among these 10 markers, Ang-2, Collagen IV, E-Cadherin, E-Selectin, IL-8, and TSP-2 were decreased on-treatment, while MMP-2, PEDF, PlGF, and VCAM-1 increased on-treatment (Fig. 1).

Within the VEGF axis, multiple family members were noted to change with treatment. VEGF-D was significantly increased (*P* = 0.001), while VEGF-C and soluble VEGF-R2 were significantly decreased (*P* < 0.05 and *P* = 0.006, respectively). Soluble VEGF-R1 was also decreased in 22 of 38 patients, although it did not reach statistical significance (*P* = 0.53).

### Correlation among biomarkers

To better understand the potential coregulation of specific biomarkers, Spearman's rank correlation was used to test pairwise correlations at baseline and on-treatment. Statistically significant pairs of baseline markers (correlation coefficients ≥0.75, *P* < 0.001) included TGF-β1 and TGF-β2, TSP-2 and collagen IV, TSP-2 and IL-8, and d-dimer and CRP (data not shown). Statistically significant pairs of on-treatment markers include TGF-β1 and TGF-β2, TSP-2 and E-Selectin, and Gro-α and IL-8. All baseline and on-treatment analyte pairs were positively correlated, indicating that biomarkers were either both high (or increased) or both low (or decreased). These correlations among baseline and on-treatment biomarkers are illustrated in dendrogram plots (Fig. S1).

### Univariate correlation of biomarkers with patient outcome

Baseline levels and on-treatment changes of analytes were next correlated with PFS and OS, the primary and secondary endpoints of the clinical study, respectively. At baseline, vWF and Ang-2 were significantly correlated with PFS (*P* = 0.0014 and 0.0347, respectively); high levels of either analyte correlated with shorter PFS (Table 3A). Baseline levels of six analytes were significantly correlated with OS: VCAM-1, vWF, TSP-2, IL-8, MMP-2, and Ang-2 (*P* < 0.05). In general, higher baseline levels for these markers were associated with shorter OS, with the only exception being MMP-2. Two analytes, vWF and Ang-2 were significantly correlated with both PFS and OS.

**Table 3:** Correlation of biomarkers with clinical outcomes: (A) biomarker baseline levels and (B) biomarker on-treatment changes

Biomarker	*P*-value	Hazard ratio	CI
(A) Baseline levels			
PFS			
vWF	0.0014	2.1	1.0–4.4
Ang-2	0.0347	1.6	0.8–3.2
OS			
VCAM-1	0.0018	2.88	1.4–6.0
vWF	0.0195	1.68	0.8–3.4
TSP-2	0.0242	1.35	0.67–2.7
IL-8	0.0304	2.2	1.06–4.4
MMP-2	0.0413	0.46	0.22–0.96
Ang-2	0.0423	2.0	0.99–4.05

(B) On-treatment changes			
PFS			
IGF-1	0.0385	1.87	1.05–5.83
OS			
Ang-2	0.018	0.41	0.20–0.86
TSP-2	0.0227	0.36	0.15–0.87
TGF-β2	0.0313	1.73	1.05–2.87

On-treatment changes in analytes were then correlated with PFS and OS. PFS was significantly correlated with changes in IGF-1 (*P* = 0.0385), with greater increases in IGF-1 levels correlating with shorter PFS (Table 3B). OS was significantly correlated with Ang-2, TSP-2, and TGF-β2 (*P* < 0.05). Greater decreases in Ang-2 and TSP-2 levels correlated with longer OS times, while a greater decrease in TGF-β2 level correlated with shorter OS time.

### Multivariate correlation of biomarkers with patient outcome

The best model for PFS using baseline analyte values that could be developed was a seven-marker signature consisting of HGF, VEGF-R1, MMP-9, Ang-2, TF, VEGF-A, and VEGF-C, although this model was not statistically significant (*P* = 0.37, and data not shown).

**Figure 2:**
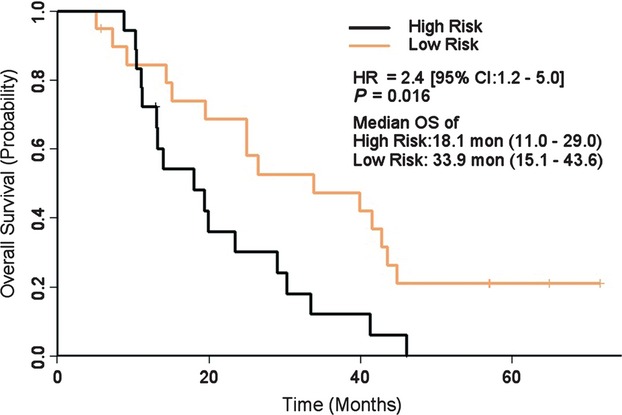
Kaplan–Meier analysis of overall survival of patients stratified according to baseline biomarkers levels. The signature for the high-risk group is baseline level of IL-6 < median; Ang-2, IGFBP-3, VCAM-1 > median.

The best model for OS using baseline analyte values consisted of a four-marker signature that included Ang-2, IGFBP-3, IL-6, and VCAM-1 (Fig. 2). The high-risk group (Ang-2, IGFBP-3, VCAM-1 > median value; IL-6 < median value) and low-risk group (Ang-2, IGFBP-3, VCAM-1 < median value; IL-6 > median value) had median survival of 18.1 and 33.9 months, respectively (*P* = 0.016).

**Figure 3:**
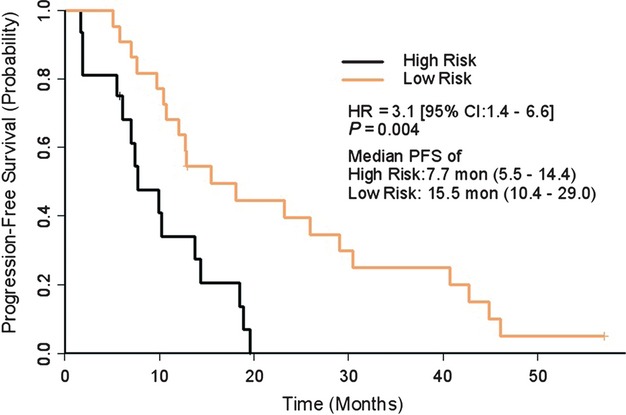
Kaplan–Meier analysis of progression-free survival of patients stratified according to on-treatment biomarker changes. The L-ratio signature for the high-risk group is IL-6, OPN, TGF-β1, E-Cadherin, MCP-1 < median; Ang-2 > median.

Next, models were developed correlating biomarker changes on-treatment with clinical outcome. For PFS, a six-marker on-treatment signature was generated (*P* = 0.004, Fig. 3). For the low-risk group (L-ratio of IL-6, OPN, TGF-β1, E-Cadherin, MCP-1 > median value; L-ratio of Ang-2 < median value) the median PFS was 15.5 months; for the high-risk group (L-ratio of IL-6, OPN, TGF-β1, E-Cadherin, MCP-1 < median value; L-ratio of Ang-2 > median value) the median PFS was 7.7 months. For OS, the optimal on-treatment signature featured two markers: Gro-α and IL-6. However, log-rank test did not reveal any survival differences in patients stratified according to this signature (*P* = 0.52, data not shown).

## Discussion

When this trial was initiated in 2003, bevacizumab had not yet been approved for CRC treatment. The study explored the efficacy and toxicity of a novel combination of bevacizumab and oxaliplatin, given every 2 weeks; and capecitabine, given on a 5-days-on, 2-days-off schedule 11.

Biomarker analyses from this study suggested an overall downregulation of multiple angiogenesis factors and the possible activation of compensatory pathways. Compared with baseline levels, 26 biomarkers were significantly changed by the end of two cycles (*P* < 0.05). Of these biomarkers, 10 were significant even after correction for multiple testings (*P* ≤ 0.0001), both by the magnitude of change and by the frequency of patients undergoing such change.

Among these biomarkers, Ang-2, TSP-2, and vWF are particularly interesting given their pivotal roles in angiogenesis and their correlation with clinical outcomes both at baseline and on-treatment. Ang-2 is overexpressed and is prognostic in a number of tumor types, including CRC 14. Ang-2 biology is known to be VEGF dependent and has been shown to be downregulated in response to anti-VEGF therapy by multiple groups, including our own 15,16. In the current study, consistent with the results of Goede et al., we found that low Ang-2 baseline levels correlated with longer PFS and OS 16. Greater reductions in Ang-2 level also predicted for better OS (Table 3A and B). The consistency of these baseline and on-treatment results suggests that Ang2 may be an important marker of benefit from anti-VEGF therapy. These findings also suggest the potential value of targeting VEGF and Ang2/Tie2 together 17. TSP-2 is an endogenous angiogenic inhibitor 18. TSP-2 was noted to cluster with multiple inflammatory mediators in this study (see Fig. S1). The reason for the downregulation of TSP-2 is unclear, yet it is consistent with observation that anti-VEGF therapy modulates both angiogenic and inflammatory responses. It should be noted that TSP-2 baseline levels as well as on-treatment changes significantly correlated with OS. Finally, vWF is a glycoprotein playing an important role in the pathogenesis of metastasis 19. High plasma vWF concentrations have been reported in various types of cancer, including CRC 20. In this study, high baseline levels of vWF were significantly correlated with both PFS and OS.

Within the VEGF axis, PlGF increased on-treatment in essentially all patients (37 of 38 patients). Upregulation of PlGF in response to anti-VEGF therapy has been noted by many groups, including our own 21,22. In addition to PlGF, statistically significant changes were also noted for sVEGF-R2 (*P* = 0.0055) and VEGF-D (*P* = 0.0011). Soluble VEGF-R2 was consistently downregulated in 24 of the 38 patients in this study. Reduction in soluble VEGF-R2 in response to anti-VEGF therapy has been reported by others and is likely mechanism-based 23. VEGF-D level increased on-treatment in 30 of 38 patients, potentially as a compensatory response to VEGF-A and/or VEGF-R2 inhibition. Interestingly, our analysis of CALGB80303 (gemcitabine ± bevacizumab in advanced pancreatic cancer) indicated VEGF-D as a potential predictive marker for benefit from bevacizumab, where low levels predicted for benefit from bevacizumab and high levels predicted for lack-of-benefit from bevacizumab 10. Weickhardt et al. 24 also found that lower expression of VEGF-D in tumor tissue was associated with greater PFS and OS benefit from bevacizumab in mCRC patients. In the current study, however, VEGF-D was not significantly correlated with PFS or OS, possibly due to differences in biology, the small sample size, and/or the potential for prognostic and predictive markers to be confounded in nonrandomized studies.

Our analysis of more than 40 biomarkers enabled us to study concurrent regulation of multiple pathways. From pairwise correlations and dendrograms, extensive cross-talk among inflammatory cytokines, growth factors, and matrix derived angiogenic factors and modulators were noted. The most significant correlation was detected for TGF-β1 and TGF-β2, which appeared to be highly coregulated both at baseline and during the course of treatment.

Combining multiple angiogenic biomarkers to generate diagnostic, prognostic, and predictive signatures is a relatively novel, yet highly promising strategy. Zurita et al. defined a six-marker baseline biomarker signature for the multi–tyrosine kinase inhibitor sorafenib (osteopontin, VEGF, carbonic anhydrase 9, collagen IV, VEGF-R2, and tumor necrosis factor-related apoptosis-inducing ligand), which was able to select metastatic renal cell carcinoma patients with the greatest PFS benefit 25.

Similarly, our work developed potential models to predict clinical benefit, featuring a four-marker baseline model for OS, and a six-marker on-treatment model for PFS. We used these models to divide patients into high- and low-risk groups. Kaplan–Meier plots showed significant clinical benefit for the low-risk groups for both OS and PFS (Figs. 2 and 3). These models merit further evaluation in larger, randomized trials.

Using a novel proprietary assay, Jayson et al. 26,27 have reported that a VEGF-A assay that preferentially binds small VEGF-A isoforms may predict for benefit from bevacizumab in metastatic breast, pancreatic, and gastric cancers. However, VEGF-A levels in that assay were not predictive of benefit from bevacizumab in colorectal, non-small cell lung, or renal cell cancers, perhaps due to preanalytic issues with those studies. Our analysis of phase III data from CALGB80303 also identified several markers with significant prognostic importance, as well as markers that may predict for benefit or lack of benefit from bevacizumab. Taken together, results from multiple phase III studies support the use of angiome analyses to identify potentially useful prognostic and predictive markers.

The results from the current phase II study are limited by the small sample size and nonrandomized nature of the study. In nonrandomized studies, markers associated with general prognosis versus markers that predict for benefit from a specific treatment cannot be distinguished and may interact and confound the analysis of each other. Although many of our results were highly statistically significant, they should nevertheless be considered exploratory and hypothesis generating. While treatment related changes use each patient as their own control, it is not yet clear whether these changes are driven primarily by bevacizumab, by the capecitabine/oxaliplatin chemotherapy, or by the tumor's or host's response to these agents. However, the treatment-related changes seen in the current study are consistent with multiple reports with various anti-VEGF agents and appear to describe an anti-VEGF class effect on PlGF, Ang-2, and sVEGF-R2. The treatment related changes we noted in TSP-2 and several other markers (IGF-1, TGF-β2) have not been broadly reported and deserve further exploration. Intriguingly, univariate and multivariate analyses from the current study, both at baseline and on-treatment, appear to be consistent with our findings in CALBG80303, suggesting a potential role for VEGF-D, Ang-2, and multiple inflammatory factors, as prognostic and/or predictive markers for anti-VEGF therapy.

In summary, our plasma angiome analysis in this phase II study identified multiple angiogenic markers with potential prognostic and/or predictive importance for CRC patients treated with bevacizumab in combination with capecitabine/oxaliplatin. These results merit follow-up randomized studies in the future.

## Supporting Information

Figure S1 Dendrogram plots of hierarchical clustering patterns of biomarkers for both baseline (A) and on-treatment (B).
