# Supplementary material for: Correlation of angiogenic biomarker signatures with clinical outcomes in metastatic colorectal cancer patients receiving capecitabine, oxaliplatin, and bevacizumab
Source: Cancer Med. 2013 Mar 6;2(2):234–42. doi: 10.1002/cam4.71 (PMC3639662; doi:10.1002/cam4.71)

Supplement figure 1. Dendrogram plots of hierarchical clustering patterns of biomarkers for both baseline (A) and on-treatment (B).

A. Baseline.


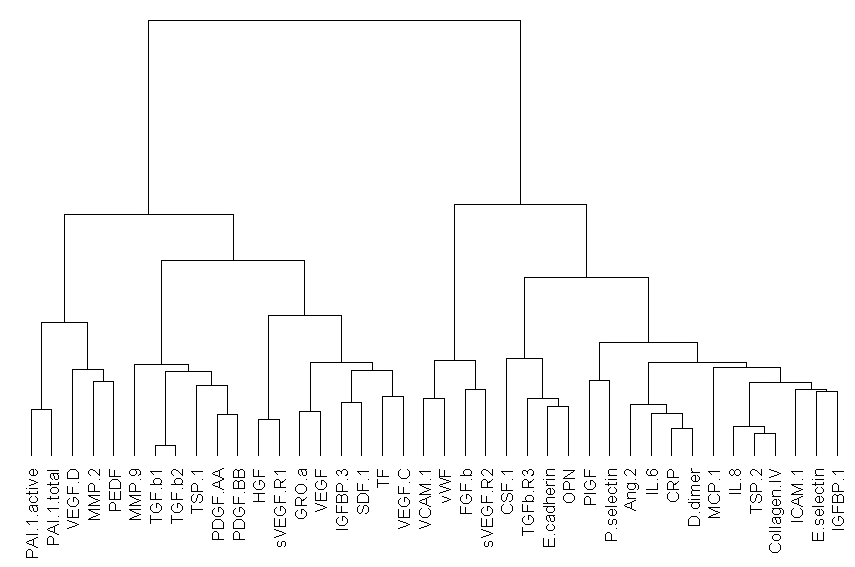


B. On-treatment.


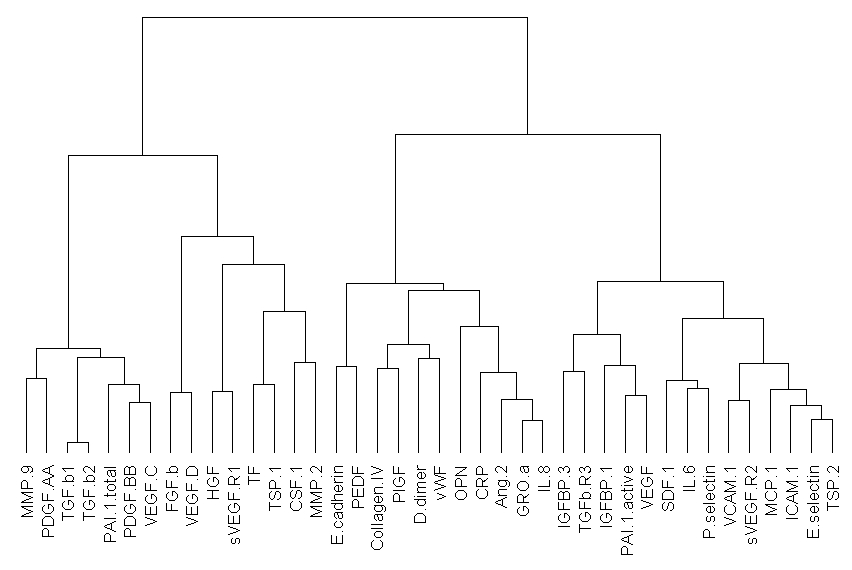

Supplement: Figure S1 — Dendrogram plots of hierarchical clustering patterns of biomarkers for both baseline (A) and on-treatment (B). [file cam40002-0234-sd1.docx]
